# Whole-Genome Sequencing and Functional Characterization of a Novel *Kuravirus* Bacteriophage with Antibiofilm Activity Against Multidrug-Resistant Avian Pathogenic *Escherichia coli*

**DOI:** 10.3390/ijms262411911

**Published:** 2025-12-10

**Authors:** Phitchayapak Wintachai, Renuka Thonguppatham, Martha R. J. Clokie, Thotsapol Thomrongsuwannakij

**Affiliations:** 1 Bacteriophage Laboratory, Walailak University, Thasala, Nakhon Si Thammarat 80161, Thailand; renuka.th@mail.wu.ac.th; 2 School of Science, Walailak University, Thasala, Nakhon Si Thammarat 80161, Thailand; 3 Becky Meyer Centre for Phage Research, Department of Genetics, Genomics, and Cancer Sciences, University of Leicester, Leicester LE1 7RH, UK; mjrc1@le.ac.uk; 4 Akkhraratchakumari Veterinary College, Walailak University, Thasala, Nakhon Si Thammarat 80161, Thailand; thotsapol.th@wu.ac.th

**Keywords:** bacteriophage, avian pathogenic *Escherichia coli*, *Kuravirus*, biofilms

## Abstract

Avian pathogenic *Escherichia coli* (APEC) infections cause substantial economic losses in the poultry industry, primarily due to high mortality rates, reduced productivity, and increased treatment costs. With the emergence of antibiotic-resistant APEC strains, including multidrug-resistant (MDR) variants, alternative therapeutic strategies have gained increasing attention. This study reports the isolation and characterization of an *Escherichia* phage, vB_EcoG_APECPW12 (phage vAPECPW12), which specifically targets MDR APEC. Both antibacterial and antibiofilm activities of the phage were evaluated. Phage vAPECPW12 produced small plaques with halos and exhibited strong lytic activity against MDR APEC. Whole-genome sequencing revealed a genome size of 77,812 base pairs with 123 open reading frames. No tRNA, antibiotic-resistant, or lysogenic genes were identified. Phylogenetic analysis and genome comparison suggest that phage vAPECPW12 is a novel member of the genus *Kuravirus* within the *Gordonclarkvirinae* family. It also demonstrated good stability across a range of temperatures and pH levels and remained viable after exposure to UV radiation. Phage vAPECPW12 showed a high adsorption rate, a short latent period of 10 min, and a burst size of 258 plaque-forming units per cell. A depolymerase domain was identified in the genome, prompting investigation of its antibiofilm efficacy. Results showed that vAPECPW12 significantly inhibited biofilm formation and removed preformed biofilms, indicating its potential as an alternative antimicrobial agent for controlling MDR APEC and their biofilms in poultry farming.

## 1. Introduction

Avian pathogenic *Escherichia coli* infection in chickens is generally reported as a pathogen associated with significant morbidity and mortality in the poultry industry worldwide [1]. Avian species can be infected with avian pathogenic *E. coli* (APEC), an extraintestinal pathotype of *E. coli,* through the respiratory tract or via oral, nasal, or cloacal routes, resulting in airsacculitis, polyserositis, perihepatitis, cellulitis, coligranuloma, pericarditis, egg peritonitis, salpingitis, omphalitis, osteomyelitis/arthritis, and septicemia [2,3]. These infections are associated with a reduction in animal wellness [4]. Moreover, the infections also lead to reduced egg and meat production, hatching rate, live body weight, and feed conversion efficiency, resulting in economic losses [5]. Interestingly, APEC strains share similarities with *E. coli* strains that cause urinary tract infection, newborn meningitis, and sepsis in humans. Moreover, APEC has recently been reported as a possible foodborne zoonotic pathogen due to its ability to cause extraintestinal infections in humans [2,6]. To treat APEC infection in poultry, antibiotics are commonly administered. However, the emergence and increasing prevalence of antibiotic-resistant APEC strains such as those resistant to aminoglycosides, doxycycline, fluoroquinolones, macrolides, penicillin, and tetracyclines, including carbapenems, which are considered last-resort antibiotics, have been widely reported, raising concerns about the effectiveness of conventional treatments and the need for alternative therapeutic strategies [2,7,8]. Consequently, alternative treatments for APEC control are of interest for further development.

Bacteriophages, also known as phages, have garnered attention as a promising alternative to antibiotics. These bacterial viruses are abundant in diverse environments and can specifically infect and lyse target bacteria. Their high host specificity and minimal disruption to the normal microbiota of animals and humans make phages an attractive option for combating bacterial infections. Numerous phages targeting APEC have been reported as potential therapeutic agents, including *Escherichia* phage PEC9 from China [9], *Escherichia* phages SKA49 and SKA64 from Pakistan [10], *Escherichia* phage AG- MK-2022 Basu from Iran [11], and phage vAPECPW08 from Thailand [12]. However, isolation and characterization of new phages specific to locally circulating bacterial pathogens remain essential due to the ongoing evolution of bacterial pathogens.

In this study, we aimed to isolate and characterize a virulent phage specific to an APEC strain. The fundamental characteristics of the phage, its whole-genome analysis, and its antibacterial and antibiofilm activities were evaluated in this study.

## 2. Results

### 2.1. Isolation and General Features of Phage

**Figure 1:**
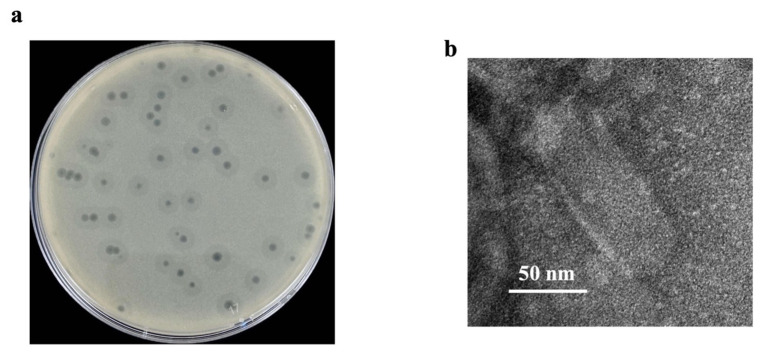
Plaque formation and morphology of *Escherichia* phage vAPECPW12; (**a**) Plaques formed by phage vAPECPW12 on a lawn of MDR APEC strain PW007; (**b**) Transmission electron microscopy image of phage vAPECPW12 captured at 120,000× magnification.

*Escherichia* phage vAPECPW12 was isolated from an environmental water sample using APEC PW007 as a host. The phage produced small plaques (0.2–0.4 cm in diameter) surrounded by a halo zone (Figure 1a). Transmission electron microscopy (TEM) revealed that the phage possessed a prolate head with a length ranging from 119.44 to 130.56 nm and a width from 50 to 55.56 nm (Figure 1b). This morphology resembles the unusual C3 podovirus morphotype, which is characterized by an elongated or cigar-shaped capsid with an extremely short tail, as previously described [13,14].

### 2.2. Host Range Determination

The host range of *Escherichia* phage vAPECPW12 was evaluated using 25 MDR APEC isolates and 5 *Campylobacter jejuni* isolates. As shown in Table 1, phage vAPECPW12 could lyse 8 out of 25 APEC isolates but not any *C. jejuni* isolates.

### 2.3. Efficiency of Plating (EOP) of Phage

Eight MDR APEC isolates that were sensitive to *Escherichia* phage vAPECPW12 in the host range analysis were selected for the EOP assay. The results indicated that the phage exhibited high activity against 3 isolates, moderate activity against 4 isolates, and low activity against 1 isolate (Table 1).

### 2.4. Genome Characterization of Phage

**Figure 2:**
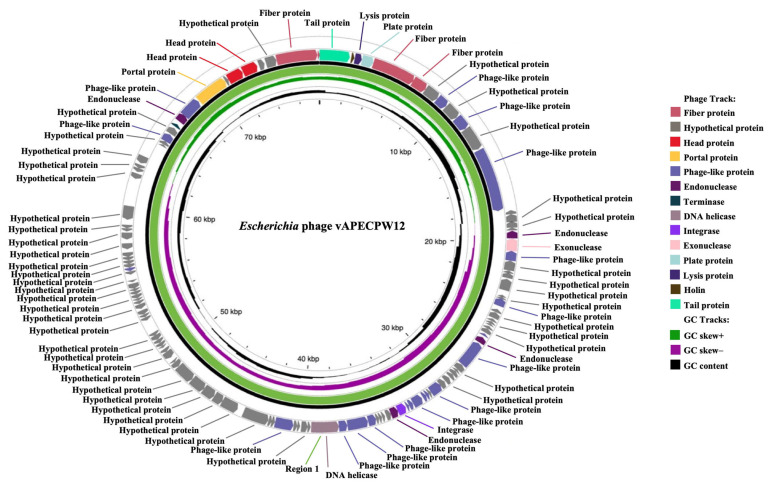
Genomic circular map of *Escherichia* phage vAPECPW12 generated with the PHASTEST web server. The CDSs were represented in different colors based on functional categories. The innermost black circle illustrates the GC content, while the purple circle denotes negative GC skew, and the green indicates positive GC skew.

The genome of *Escherichia* phage vAPECPW12 was sequenced using the Illumina HiSeq system. A total of 26,736,720 raw reads were generated, corresponding to 4,037,244,720 total read bases (Supplementary Table S1). The filtered data statistics were summarized (Supplementary Table S2). The overall quality of the raw sequencing data was assessed using the FastQC base quality plot (Supplementary Figure S1). De novo assembly was performed using SPAdes, which resulted in a single contig of 77,812 bp (Supplementary Figure S2a,b). Contig statistics are provided in Supplementary Table S3. The complete genome of phage vAPECPW12 is a circular double-stranded DNA molecule with a length of 77,812 base pairs (Figure 2). Regarding base composition, the number of adenine, thymine, guanine, and cytosine were 22,478, 22,630, 15,975, and 16,729, respectively. The G+C content of the genome was 42.03%. Assembly validation showed 100% coverage with an average depth of 165.17×.

Genome annotation was performed using the PHASTEST web server (Supplementary Table S4). The genome of phage vAPECPW12 contains 123 coding sequences (CDS), which were classified into functional categories based on their predicted roles. These include head proteins, such as the capsid decorating protein and major coat protein; tail proteins, including the tail spike and tail protein; and membrane-associated proteins, such as putative proteins containing one, two, or four transmembrane helical domains. The genome also encodes lysis-related proteins, including holin, Rz/Rz1 spanin, and N-acetylmuramidase. Additional structural components include fiber and structural proteins (tail fiber and tape measure protein), as well as DNA packaging proteins, such as DNA helicase, portal protein, and the large and small subunits of terminase. Several phage-associated proteins were identified, including RNA polymerase subunits (RNAP1 subunit B and RNA polymerase 2 subunit), 2 putative helical domain containing proteins, putative peptidoglycan binding domain containing protein, putative DNA processing chain, ADP-ribosylglycohydrolase, RNA polymerase RNAP1 subunit B, ATPase, metallopeptidase, deoxycytidine triphosphate deaminase, thymidylate synthase, rIIA-like protein, rIIB-like protein, triphosphate pyrophosphohydrolase, DNA polymerase, PD-(D/E)XK nuclease superfamily protein, DNA primase, nucleoside triphosphate hydrolase, single-stranded DNA-binding protein, virion RNA polymerase, and 2 structural proteins. The genome also encodes a cross-over junction protein, namely Holliday junction resolvase. In addition, 39 proteins were annotated as hypothetical proteins, for which specific functions could not be assigned. The predicted CDSs were further validated using BLASTp (version 2.17.0) to confirm their putative functions (Supplementary Table S5). These genes were grouped into biological roles, including genes related to structural components, DNA packaging, DNA metabolism, and replication, as well as several genes with unknown or hypothetical functions. No tRNA genes, transposons, or antibiotic resistance genes were detected in the genome. The lifestyle of phage vAPECPW12 was predicted to be virulent, based on PhageScope and PhageLead analyses, with no temperate lifestyle-associated genes identified.

### 2.5. Phylogenetic and Comparative Genomic Analysis of Phage vAPECPW12

**Figure 3:**
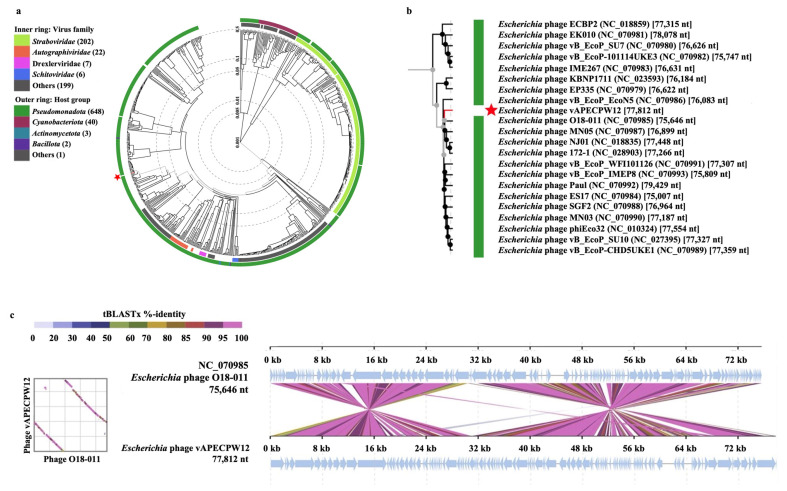
Genetic relationship between *Escherichia* phage vAPECPW12 and other phages using VipTree. (**a**) A proteomic-based phylogenetic tree generated using the complete genome of phage vAPECPW12 (indicated by a red star), compared with other phages. (**b**) A rectangular tree showing the relationship between phage vAPECPW12 and the closest relative. The red branch indicates the phage vAPECPW12 genome, whereas the black branches indicate related phage genomes retrieved from the ViPTree database. (**c**) Genome alignment between the phage vAPECPW12 genome and its closest relative, phage O18-011. Light blue arrows indicate the open reading frames. The color bands between the genome maps represent the percentage of identity between each sequence, as indicated in the legend at the top of the figure.

**Figure 4:**
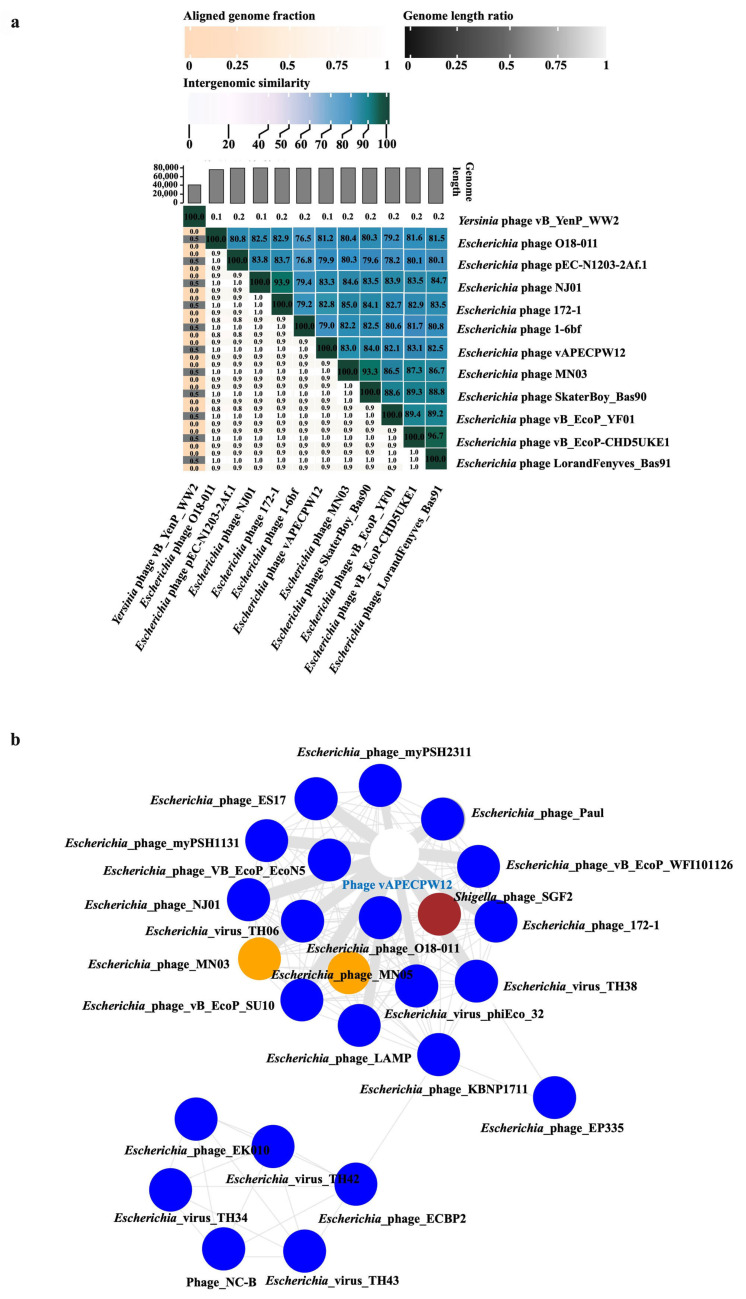
Analysis of the novelty of phage vAPECPW12. (**a**) Heat map generated by VIRIDIC showing genome similarity among 10 related phages and one outgroup phage. The right panels present intergenomic similarities using a blue gradient, with darker shades indicating higher similarity. The left panel displays three metrics: aligned genome fraction for the row genome (top), genome length ratio (middle), and aligned genome fraction for the column genome (bottom). (**b**) Novelty of phage vAPECPW12 confirmed using the PhageClouds database.

The relationship between *Escherichia* phage vAPECPW12 and other phages was initially assessed using BLASTn (version 2.17.0) in the NCBI database. The results revealed a high similarity to members of the genus *Kuravirus* within the *Gordonclarkvirinae* family. To further elucidate the evolutionary relationship, a proteomic tree was constructed using the ViPTree server (Figure 3a). This analysis indicated that the phage is associated with the bacterial host class *Pseudomonadota*. A rectangular phylogenetic tree constructed using ViPTree showed that phage vAPECPW12 is most closely related to *Escherichia* phage O18-011 (NC_070985), with both phages sharing a common evolutionary ancestor (Figure 3b). Whole-genome alignment of the phage vAPECPW12 genome and the phage O18-011 genome highlighted regions of similarity and divergence (Figure 3c). TaxMyPhage was employed to evaluate genome similarities and assign taxonomic classification [15]. The results indicated that phage vAPECPW12 belongs to the genus *Kuravirus*, family *Gordonclarkvirinae*, order *Caudoviricetes*, phylum *Uroviricota*, kingdom *Heunggongvirae*, and realm *Duplodnaviria*. Thus, the phage was named as phage vB_EcoG_APECPW12 according to the binomial nomenclature of bacterial viruses [16]. To identify its novelty, the Virus Intergenomic Distance Calculator (VIRIDIC) was used to calculate intergenomic similarities among phage genomes (Figure 4a) [17]. The nucleotide identities between phage vAPECPW12 and closely related phages range from 82.1% to 83.1%, supporting its classification as a novel member within the genus *Kuravirus*. Moreover, the novelty of the phage was further confirmed through analysis using the PhageClouds database (Figure 4b) [18]. The result indicates that phage vAPECPW12 is a new member in the genus *Kuravirus*, family *Gordonclarkvirinae*, order *Caudoviricetes*, phylum *Uroviricota*, kingdom *Heunggongvirae*, and realm *Duplodnaviria*.

### 2.6. Adsorption Rate of Phage

**Figure 5:**
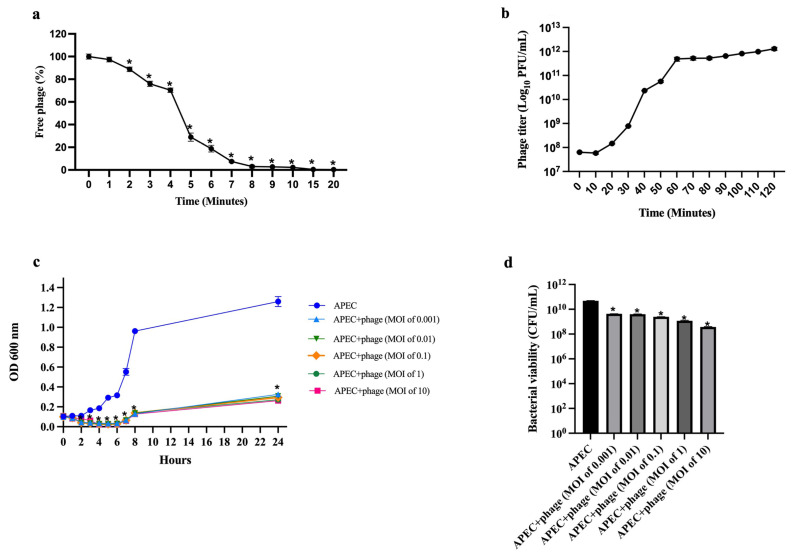
Biological characterization of *Escherichia* phage vAPECPW12; (**a**) adsorption efficacy of phage vAPECPW12 to its host cells; (**b**) one-step growth curve of phage vAPECPW12 on its host cells; (**c**) bacterial growth inhibition measured by OD_600_ every hour from 0 to 8 h and at 24 h; (**d**) bacterial cell viability following phage vAPECPW12 treatment at various MOIs, assessed by colony forming unit (CFU) counts at 24 h. Statistical analysis was performed using one-way ANOVA with Dunnett’s post hoc test. Bars represent the standard error of the mean (SEM), and asterisks indicate significant differences (* *p* ≤ 0.05).

Figure 5a presents the adsorption efficacy of *Escherichia* phage vAPECPW12 to its host cells. The number of free phages decreased as incubation time increased. At 6 min post-incubation, over 80% of the phages had adsorbed to the bacterial cells, and by 20 min, nearly 100% adsorption was observed. The adsorption rate constant (*k*) for phage vAPECPW12 was calculated to be 5.63 × 10^−9^ mL/min.

### 2.7. Growth Characteristics of Phage

A one-step growth curve was conducted to assess the replication dynamics of *Escherichia* phage vAPECPW12 (Figure 5b). The phage exhibited a latent period of 10 min. Following this phase, the titer increased steadily and reached a plateau at approximately 60 min. The burst size was approximately 258 plaque-forming units (PFU)/cell.

### 2.8. Lytic Activity of Phage

The inhibition effect of *Escherichia* phage vAPECPW12 on APEC was evaluated at MOIs ranging from 0.001 to 10. The optical density of untreated APEC cultures at 600 nm (OD600) continuously increased, indicating active bacterial growth (Figure 5c). In contrast, the OD_600_ values of APEC cultures treated with phage vAPECPW12 at all tested MOIs were significantly reduced within 1 h of incubation, indicating rapid bacterial lysis. However, a gradual increase in OD_600_ was observed at 7 h post-incubation in the cultures treated with all MOIs, suggesting partial regrowth or recovery of the bacterial population. Nevertheless, the OD_600_ values in all phage-treated groups remained consistently lower than those of the untreated control throughout the experiment, at 24 h post-incubation. Bacterial viability assay was determined to confirm the lytic effect of phage vAPECPW12. Treatment at MOIs of 0.001 to 10 significantly reduced viable bacterial counts by approximately 1.06 to 2.13 log units, respectively, compared to the untreated control (Figure 5d). These findings indicate that higher phage concentrations effectively reduced bacterial growth (OD_600_) and viability.

### 2.9. Morphology of Bacterial Cells After Phage Treatment Under Electron Microscopy

**Figure 6:**
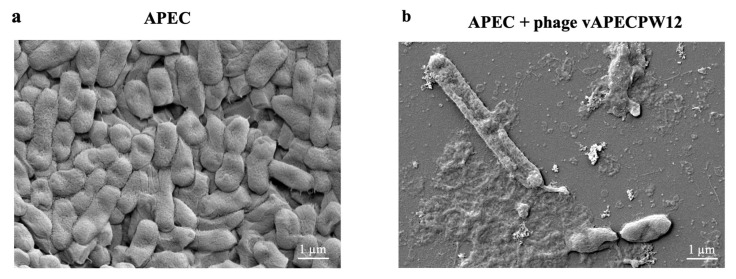
Effect of *Escherichia* phage vAPECPW12 on the morphology of APEC cells. (**a**) Untreated APEC cells; (**b**) APEC cells treated with phage vAPECPW12. Images were captured at 10,000× magnification.

Morphological alterations in APEC cells following *Escherichia* phage vAPECPW12 treatment were observed using a scanning electron microscope (SEM) and compared with untreated control cells. The untreated APEC cells exhibited a typical rod-shaped morphology with a smooth and intact surface (Figure 6a). In contrast, APEC cells treated with phage vAPECPW12 showed clear signs of lysis (Figure 6b). Membrane damage, including wrinkles and pore formation, was evident, leading to cell lysis and bacterial death. Numerous cell debris fragments, indicative of bacterial lysis, were observed in the phage-treated samples.

### 2.10. Phage Temperature, pH, and Ultraviolet (UV) Radiation Stability

**Figure 7:**
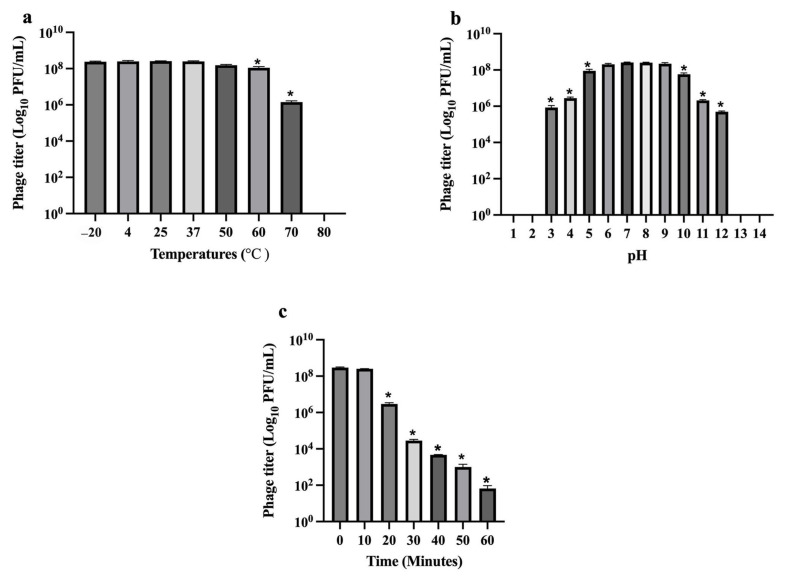
Effect of physical and chemical agents on the stability of *Escherichia* phage vAPECPW12. The stability of phage vAPECPW12 was evaluated under the following conditions: (**a**) various temperatures (−20 °C to 80 °C), (**b**) a range of pH values (pH 1–14), and (**c**) exposure to ultraviolet (UVC) light for 0 to 60 min. Statistical analysis was performed using one-way ANOVA with Dunnett’s post hoc test. Bars represent the standard error of the mean (SEM), and asterisks indicate significant differences (* *p* ≤ 0.05).

The stability of *Escherichia* phage vAPECPW12 was evaluated under varying temperatures, pH, and UV radiation conditions. Phage titers remained stable after a 2 h incubation at −20 °C, 4 °C, 25 °C, 37 °C, and 50 °C (Figure 7a). However, a significant reduction in phage titer was observed at 60 °C and 70 °C, and no phage viability was detected at 80 °C. Regarding pH stability, phage vAPECPW12 remained stable within the pH range of 6 to 9 after a 2 h incubation (Figure 7b). A significant decrease in phage viability was observed at pH 3, 4, 5, 10, 11, and 12. No viable phage was detected at extreme pH values of 1, 2, 13, and 14. The effect of UV radiation on phage stability was also assessed (Figure 7c). The phage titer was reduced by nearly 2 log units within 20 min of UV exposure. Nevertheless, the phage titer remained detectable after 60 min of exposure.

### 2.11. Efficacy of Phage to Remove Preformed Biofilms

**Figure 8:**
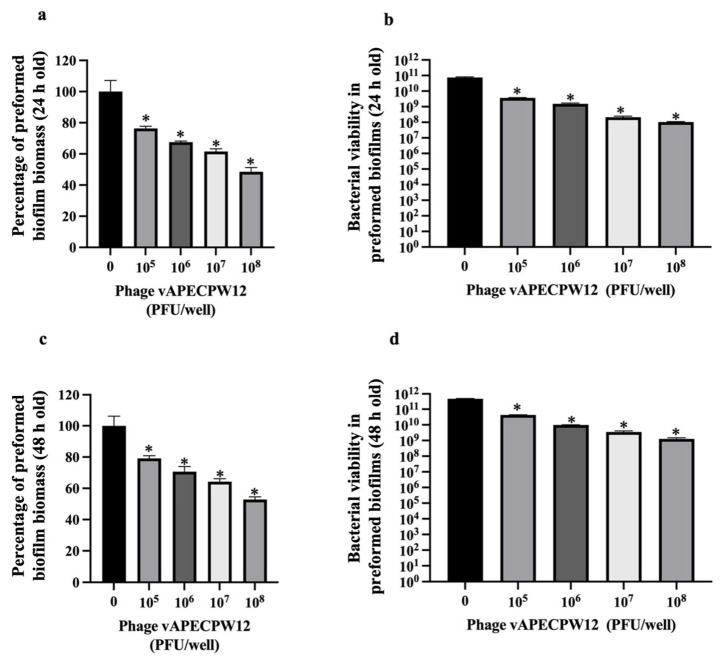
Efficacy of *Escherichia* phage vAPECPW12 in removing preformed biofilms. APEC biofilms were treated with phage vAPECPW12 at concentrations ranging from 10^5^ to 10^8^ PFU/mL, followed by determination of biofilm biomass and bacterial viability. For 24 h old biofilms, biofilm biomass and bacterial viability are shown in panels (**a**) and (**b**), respectively. For 48 h old biofilms, panels (**c**) and (**d**) show biofilm biomass and bacterial viability, respectively. Statistical analysis was performed using one-way ANOVA with Dunnett’s post hoc test. Error bars represent the standard error of the mean (SEM), and asterisks indicate statistically significant differences (* *p* ≤ 0.05).

The ability of *Escherichia* phage vAPECPW12 to remove both 24 h and 48 h preformed biofilms was investigated by measuring biofilm biomass and counting colony-forming units (CFUs). Treatment with phage vAPECPW12 at 10^5^ to 10^8^ PFU/mL significantly decreases the biofilm biomass and bacterial viability of 24 h preformed biofilms by approximately 23.62 to 51.49% and 1.39 to 2.65 log units, respectively (Figure 8a,b). For 48 h preformed biofilms, phage vAPECPW12 at 10^5^ to 10^8^ PFU/mL significantly decreases the biofilm biomass and bacterial viability by approximately 20.85 to 47.15% and 1.3 to 2.33 log units, respectively (Figure 8c,d). The highest concentration of phage, at 10^8^ PFU/mL, showed the greatest reduction.

### 2.12. Effectiveness of Phage to Reduce Biofilm Formation

**Figure 9:**
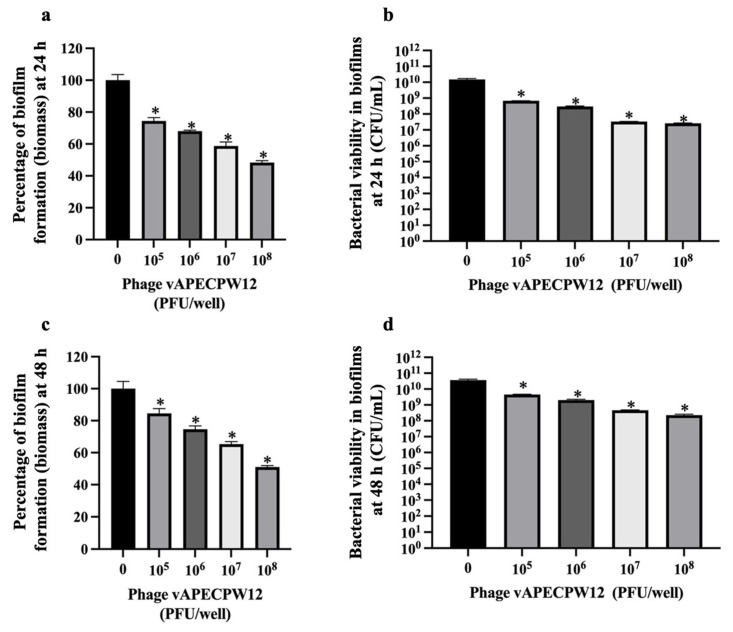
Efficacy of *Escherichia* phage vAPECPW12 in reducing biofilm formation. APEC cells were incubated with phage vAPECPW12 at 10^5^ to 10^8^ PFU/mL. At 24 and 48 h post-incubation, biofilm biomass and bacterial viability were assessed. In the case of 24 h old biofilms, panels (**a**) and (**b**) display the biofilm biomass and bacterial viability, respectively. For 48 h old biofilms, panels (**c**) and (**d**) show biofilm biomass and bacterial viability, respectively. Statistical analysis was performed using one-way ANOVA with Dunnett’s post hoc test. Error bars represent the standard error of the mean (SEM), and asterisks indicate statistically significant differences (* *p* ≤ 0.05).

The ability of *Escherichia* phage vAPECPW12 to reduce biofilm formation was assessed at 24 h and 48 h post-incubation. At 24 h, treatment with phage vAPECPW12 at concentrations ranging from 10^5^ to 10^8^ PFU/mL resulted in a 25.57 to 51.66% reduction in biofilm biomass and a 1.47 to 2.89 log CFUs/mL decrease in bacterial viability (Figure 9a,b). At 48 h post-incubation, phage treatments at the same concentration range led to a significant reduction of 15.4 to 48.96% in biofilm biomass, and 0.92 to 2.14 log CFUs/mL in bacterial viability (Figure 9c,d). Among all concentrations tested, 10^8^ PFU/mL demonstrated the most significant reduction in biomass and viable cell counts.

## 3. Discussion

MDR infections in animals and humans have become important problems worldwide. When MDR bacteria infect, it is very difficult to control and reduce the infection because these bacteria are resistant to commonly used antibiotics. The focus on infections in poultry farms and industries highlights that the extensive and improper use of antibiotics in the poultry sector has contributed significantly to the emergence of antibiotic-resistant APEC strains, which impact animal health and food safety. Resistance to almost all antibiotics has been reported, including resistance to polymyxins, which are the last resort against Gram-negative bacteria [2]. Thus, the emergence of MDR APEC highlights the need for new strategies to combat their infections. Nowadays, various alternative treatments, including plant-derived antimicrobials, antimicrobial peptides, nanoparticles, probiotics, microbiota modulation, antibodies, and enzymes, have been developed as potential alternatives. Phage therapy has been valuable due to its efficacy and specificity.

In this study, *Escherichia* phage vAPECPW12 was successfully isolated. The phage produced small, clear plaques with halos, suggesting potential depolymerase activity. Phage vAPECPW12 lysed 8 out of 25 tested APEC isolates (32%) but showed no activity against *C. jejuni*, which has been reported as a highly prevalent bacterium in chickens [19]. A study from the region where our isolates were obtained, in the southern part of Thailand, also reported that *C. jejuni* is more prevalent than APEC [20]. The results indicate that phage vAPECPW12 may be specific to APEC, thereby reducing the risk of disrupting the niche microbiota [21]. Further investigation of its host specificity would be beneficial. When comparing the host range of phage vAPECPW12 with those of other reported phages, phage vAPECPW12 exhibited a narrower host range than phage SKA49 (71.43%), SKA64 (33.33%), and AG-MK-2022 (57.1%) [10,22]. However, the host range of phage vAPECPW12 was broader than that of several phages targeting APEC isolates, including phage UPEC04 (17.9%), UPEC6 (10.7%), UPEC10 (10.7%), UPEC03 (8.9%), UPEC01 (7.1%), UPEC08 (7.1%), and UPEC09 (1.8%) [23]. The phylogenetic groups of the APEC isolates used in this study were previously reported, with most belonging to phylogroups D (56%) and B2 (44%) [24]. The bacterial host used for phage isolation in this study was classified as phylogroup D. The phage lysed isolates from both phylogenetic groups. However, phylogenetic grouping could not be used to determine the host-range specificity of the phage. Previous studies reported that phylogenetic groups D and B2 are significantly associated with the K1 capsule biosynthesis gene cluster [25]. Moreover, an Endo-N-acetylneuraminidase-like domain was detected in the phage genome, and this domain has been reported to be required for phage penetration into the *E. coli* K1 capsule [26]. The results and previous studies could support the hypothesis that phage vAPECPWPW12 might be specific to the K1 capsule. However, genome sequencing of the bacterial isolates used in this study should be prioritized in future investigations to clarify this hypothesis. Given the current host range of phage vAPECPW12, its spectrum of activity appears relatively narrow. Further studies using a larger and more diverse collection of APEC isolates would help clarify the breadth of the host range [27], and cross-serogroup assessment should be included in future evaluations. Additional studies assessing the potential application of phage vAPECPW12 under on-farm conditions would strengthen the understanding of its practical usefulness.

The genome of phage vAPECPW12 is 77,812 bp, circular, and double-stranded DNA, within the size range of reported APEC phages (39,917 to 166,374 bp) [9,10,12,28,29]. Annotation revealed genes grouped into 4 major functional modules: structure, replication/regulation, lysis, and packaging. No tRNA, antibiotic-resistant genes, transposon-related genes, or integrase genes were detected, suggesting the phage is safe for applications. Notably, CDSs at positions 2191 to 2409, 2439 to 2930, and complement 21,764 to 21,937 were predicted to encode holin, endolysin, and o-spanin, respectively, key host cell lysis enzymes. Holin forms pores in the inner membrane, allowing endolysin to access and degrade the peptidoglycan layer, while spanins disrupt the outer membrane in Gram-negative bacteria, completing the lysis process [30,31,32]. Complete lysis enables the release of new phage progeny from infected bacterial cells. Comparative genomics confirmed that phage vAPECPW12 belongs to the genus *Kuravirus*, within the *Gordonclarkvirinae* family, *Caudoviricetes* order, *Uroviricota* phylum, *Heunggongvirae* kingdom, and *Duplodnaviria* realm. Nucleotide identity analysis indicated that phage vAPECPW12 is a novel member of the genus *Kuravirus*. Its lytic lifestyle, as predicted by PhageScope and PhageLead, makes it favorable for therapeutic use, as lytic phages do not integrate their genomes into the bacterial host genome, thereby reducing the risk of gene transfer and associated increases in bacterial virulence [33,34].

Stability under environmental stressors, such as temperature, pH, and UV exposure, is critical for phage applications. Phage vAPECPW12 remained stable across a broad temperature and pH range, supporting its utility in diverse conditions. Its tolerance to various temperatures indicates that phage vAPECPW12 is suitable for storage and transportation. Moreover, its stability across a broad pH range suggests that it could maintain activity in the acidic environments of the proventriculus and gizzard, allowing it to reach the intestines, where APEC commonly colonize. While UV exposure reduced phage activity as expected, lytic activity remained detectable, indicating partial UV resistance.

Phage adsorption efficiency strongly influences its infection potential. Rapid adsorption, in which over 80% of phage vAPECPW12 adsorbed to the bacterial cell within 6 min, allowed the phage to bind, penetrate, and initiate the infection quickly, thereby enhancing bacterial control [28,35,36]. Phage vAPECPW12 exhibited more than 90% within 10 min, outperforming previously reported APEC phages, such as PEC9 [9], and vECPW8 [12]. Once inside the host, phages with shorter latent periods replicate more rapidly, resulting in quicker production and release of progeny phage particles from the infected bacteria. Phage vAPECPW12 had a shorter latent period than *Escherichia* phages SKA49 (35 min), SKA64 (30 min), and vECPW8 (20 min), suggesting faster replication. It also had a higher burst size compared to PEC9, vECPW8, and AG-MK-2022 Basu, which had burst sizes ranging from 68 to 152 PFU/mL, indicating that more progeny phages were produced per infected cell [9,12,22]. These properties enhance its suitability for therapeutic development. While bacterial resistance to the phage was observed after 10 h, bacterial growth remained lower than in untreated controls. Although bacterial growth appeared visually similar across MOI treatments at the end of the experiment, statistical analysis revealed significant differences among the MOIs. Notably, previous studies have also reported minimal variation in cultures treated with vastly different MOIs toward the end of the experiment, such as *Escherichia* phage vE20 (MOIs 0.00001 to 1) [37], *Enterococcus faecalis* phage SA14 (MOIs 1 to 1000) [38], and *Aeromonas hydrophila* phage AhyVDH1 (MOIs 1 and 10) [39]. Using MOIs lower than those used in this study may result in more noticeable differences in bacterial growth at intermediate time points. Approaches such as phage cocktails, engineered phage proteins, or phage training may help overcome or reduce the emergence of phage resistance [40,41,42].

The halo around plaques indicated possible depolymerase activity [43,44]. Analysis using the MPI Bioinformatics Toolkit, an interactive web service, revealed a depolymerase domain between positions 3756 and 6773. This enzyme can degrade polysaccharides found on bacterial capsules, lipopolysaccharides in bacterial outer membranes, and the exopolysaccharide (EPS) matrix of biofilms. A gene encoding Endo-N-acetylneuraminidase (Endo-N) was identified (positions 2 to 2098), classified as a tail fiber protein. Endo-N specifically degrades the polysialic acid capsule of *E. coli* and has been reported as a phage-encoded depolymerase [45]. These findings suggest that phage vAPECPW12 likely possesses antibiofilm activity. However, these explanations are based on previous reports, and the underlying mechanisms remain hypothetical. Further studies on the production of the depolymerase protein encoded by phage vAPECPW12 and the direct evaluation of its activity against biofilms would help clarify the relationship between this depolymerase and the observed antibiofilm activities.

Biofilms, a bacterial community, are major virulence factors that contribute to the persistence of infections [46,47]. Biofilms are embedded in EPS, which protects bacteria from antibiotics and environmental stress, making them difficult to control [48]. Depolymerase can degrade EPS and reduce biofilm mass. Moreover, depolymerase-mediated degradation of protective layers may increase bacterial susceptibility to immune responses and antibacterial treatment [49]. Phage vAPECPW12 effectively reduced preformed biofilms and inhibited biofilm formation, likely due to the combined action of depolymerase, holin, and endolysin encoded in its genome [49]. These enzymes degrade both the EPS matrix and bacterial cells. Combination therapies involving phage and other agents such as antibiotics or enzymes may enhance efficacy, as supported by previous studies [50,51].

## 4. Materials and Methods

### 4.1. Bacterial Strains and Growth Conditions

A total of 25 MDR APEC isolates and 5 *C. jejuni* isolates, provided by Dr. Thotsapol Thomrongsuwannakij from Akkhraratchakumari Veterinary College, Walailak University, were used in this study [24]. MDR APEC PW007 was used as the host bacterium for phage isolation and was subsequently used in all downstream experiments. All MDR APEC isolates were cultured on tryptic soy agar (TSA; Becton, Dickinson and Company, Franklin Lakes, NJ, USA). Pure colonies were then inoculated into tryptic soy broth (TSB; Becton, Dickinson and Company, Franklin Lakes, NJ, USA) and incubated at 37 °C with shaking at 150 rpm for 6 h or overnight. For *C. jejuni* cultures, the isolates were cultured on Campylobacter blood-free selective agar base (Oxoid, UK) supplemented with CCDA selective supplement (Oxoid, UK), then transferred into Mueller-Hinton Broth (MHB; Becton, Dickinson and Company, Franklin Lakes, NJ, USA) supplemented with 0.5% yeast extract (Gibco, Thermo Fisher Scientific, Waltham, MA, USA). Cultures were incubated in an anaerobic jar with a gas pack (Oxoid, UK) at 42 °C under microaerobic conditions without agitation.

### 4.2. Phage Isolation and Purification

A phage was isolated and purified following standard protocols described in earlier studies [52]. Briefly, environmental freshwater samples were collected from a pond near the poultry farm, which served as the original source of some of the bacterial isolates used in this study. The sampling site is situated in Thasala, Nakhon Si Thammarat, Thailand. The samples were cleared of all debris by centrifugation at 6000× *g* for 10 min at 4 °C. The supernatant was collected and filtered to remove some contaminated bacteria and small particles using a sterile 0.22 μm syringe filter (Merck Millipore, Burlington, MA, USA). Subsequently, the phage was enriched by incubating the filtrate with the host culture (OD600 = 0.08) at 150 rpm and 37 °C overnight. Afterward, the mixture was centrifuged at 6000× *g* for 10 min at 4 °C and filtered using a syringe filter. Plaques were examined using the double-layer agar method. Briefly, 200 µL of the diluted bacterial culture was combined with molten top agar and poured onto TSA plates. The plates were incubated overnight at 37 °C. For phage purification, individual plaques were picked using a micropipette tip and transferred into Eppendorf tubes containing 1 mL of SM buffer. After incubation overnight at 4 °C, the supernatant was used for further purification by the double-layer agar method. The purification step was repeated three times to ensure the purity [53].

### 4.3. Phage Propagation

A purified phage was used to produce a phage stock by the double-agar overlay method as previously described [52]. Semi-confluent plates were overlaid with 5 mL of SM buffer. After overnight incubation at 4 °C, the supernatant was transferred to sterile Falcon tubes and then centrifuged at 6000× *g* for 10 min at 4 °C. To remove bacterial debris, the supernatant was filtered through a sterile 0.22 μm filter and then stored at 4 °C indefinitely as the phage stock. A standard plaque assay was used to determine the phage titer (plaque-forming units per milliliter (PFU/mL)).

### 4.4. Phage Morphology Using TEM

Phage morphology was examined under TEM as described previously [52]. Three microliters of the phage sample were dropped onto a carbon-coated copper grid and stained with 2% (*v*/*v*) uranyl acetate (pH 6.7). The grid was air-dried and observed under a JEM 2010 electron microscope (JEOL, Freising, Germany) operating at 200 kV.

### 4.5. Phage Host Range

The lytic activity of the phage was evaluated against 25 MDR APEC isolates and 5 *C. jejuni* isolates using the standard spot test method [53,54]. Briefly, 200 μL of log-phase bacterial cultures (OD600 = 0.08) was mixed with molten top agar and poured onto TSA plates for APEC or onto *Campylobacter* blood-free selective agar base supplemented with CCDA selective supplement for *C. jejuni*. After the top agar solidified, 5 μL of phage solution at serial dilutions (10^1^ PFU/mL to 10^8^ PFU/mL) was spotted in triplicate on each lawn. Plates were incubated overnight at 37 °C, and the lysis zone was recorded as lytic (+) or non-lytic (−). All tests were performed independently in triplicate.

### 4.6. EOP

EOP was determined by the double-agar overlay method as previously described [55]. Bacteria isolates that showed lytic activity were selected for the EOP assay. A 10-fold serial dilution of the phage (10^1^–10^8^ PFU/mL) was mixed with 200 µL of log-phase bacteria (OD600 = 0.08) and molten soft agar, then poured onto TSA plates. After overnight incubation at 37 °C, plaques were counted, and the EOP was calculated as the average PFU on the target bacteria divided by the average PFU on the host bacteria. EOP values were classified into 4 categories: high (EOP ≥ 0.5), moderate (0.1 ≤ EOP < 0.5), low (0.001 < EOP < 0.1), and inefficient (EOP ≤ 0.001). All experiments were independently conducted in triplicate.

### 4.7. Whole-Genome Sequencing and Bioinformatic Analysis

The genomic DNA extraction, whole-genome sequencing, and de novo assembly of *Escherichia* phage vAPECPW12 were performed by Macrogen Inc. (Seoul, Republic of Korea). Genomic DNA of phage vAPECPW12 was extracted using the Maxwell RSC Tissue DNA kit (Promega, Madison, WI, USA), followed by the measurement of DNA quality and quantity. DNA sequencing libraries were prepared by random fragmentation of the DNA, followed by 5′ and 3′ adapter ligation using the Nextera XT DNA library preparation kit (Illumina, San Diego, CA, USA). Library quality was assessed before sequencing on the Illumina sequencing platform. Following sequencing, base calling and quality prediction were performed to generate raw data. Quality control of raw reads was conducted using FastQC (version 0.11.5), and de novo assembly was performed using SPAdes (version 3.15.5). Gene annotation, tRNA prediction, and genome map construction were conducted using the PHASTEST web server [56]. Additionally, ORF functions were predicted using BLASTp, and depolymerase domains or other biofilm-related domains were identified using the MPI Bioinformatics toolkit (https://toolkit.tuebingen.mpg.de/jobs/5181990, accessed on 27 August 2025).

### 4.8. Phylogenetic Analysis

To assess the genetic relationship of *Escherichia* phage vAPECPW12 and other known phages, a BLASTn search was conducted to identify closely related phages. Proteomic phylogenetic trees (both circular and rectangular) and whole-genome alignments of phage vAPECPW12 and its closest related phage were generated using the VipTree web server (accessed on 27 August 2025) [57]. To evaluate the novelty of phage vAPECPW12, whole-genome sequences of associated phages were retrieved from the NCBI database and analyzed using the TaxMyPhage platform (version 0.3.6) (accessed on 27 August 2025). Taxonomic classification at the genus and species level was determined, and percentage nucleotide identity values between phage vAPECPW12 and related phages were used to assess the novelty of the phage [15]. VIRIDIC was used to calculate intergenomic similarities among phage genomes (accessed on 27 August 2025) [17]. The PhageClouds database was used to confirm the novelty of the phage (accessed on 27 August 2025) [18].

### 4.9. Phage Adsorption Assay

Phage adsorption rate was undertaken as described previously [58,59]. Exponential-phase APEC culture (OD600 = 0.08) was infected with phage vAPECPW12 at an MOI of 1. Aliquots were collected at 1 min intervals for the first 10 min and then at 10 to 20 min intervals. Samples were filtered using a 0.22 μm pore size filter and serially diluted to determine unabsorbed phage titers by the double agar layer assay. The adsorption rate constant (*k*) was calculated using the equation: *k* (mL/min) = (2.3/B*t*) × log(P_0_/P). Where B represents the bacterial concentration (CFU/mL), *t* is the time interval (min) during which the free phage titer decreases from P_0_ to P, P_0_ is the initial concentration of free virus particles (PFU/mL), and P is the concentration at time *t*. All assays were independently performed in triplicate.

### 4.10. One-Step Growth Curve

The one-step growth curve of phage vAPECPW12 was performed as described previously [52,60]. Exponential-phase APEC cultures (OD600 = 0.08) were infected with *Escherichia* phage vAPECPW12 at an MOI of 0.1 and incubated at 37 °C for 10 min to allow adsorption. The mixture was centrifuged at 6000× *g* for 10 min at 4 °C to remove unabsorbed phages. The supernatant was discarded, and the pellet was resuspended in pre-warmed TSB. Samples were incubated at 37 °C with shaking at 150 rpm. Aliquots were collected every 10 min for a total of 120 min, filtered through a 0.22 μm syringe filter, serially diluted, and plated using the double-agar overlay method to determine phage titers. The latent period was defined as the time required for a phage to replicate within host bacterial cells. At the same time, the burst size was calculated as the average number of phage particles released per infected bacterial cell. All experiments were independently conducted in triplicate.

### 4.11. Minimum Inhibitory Multiplicity of Infection

The inhibitory effect of *Escherichia* phage vAPECPW12 on APEC growth was evaluated at various MOIs (0.001, 0.01, 0.1, 1, and 10) as described previously [52]. APEC (OD600 = 0.08) was infected with phage and incubated at 37 °C with shaking at 150 rpm. Uninfected cultures served as the control. Samples were collected hourly from 0 h to 8 h for OD_600_ measurement using a UV-spectrophotometer. At 24 h post-infection, the samples were collected to measure bacterial growth by OD_600_ measurement. Moreover, bacterial viability was assessed by serial dilution and spot-plating on TSA plates. The plates were incubated overnight, and bacterial colonies were counted to calculate CFU/mL. All experiments were independently conducted in triplicate.

### 4.12. Bacterial Morphology After Phage Treatment Under SEM

Bacterial morphology following phage treatment was assessed by SEM as previously reported [12,61]. Exponential-phase APEC (OD600 = 0.08) was incubated with *Escherichia* phage vAPECPW12 at an MOI of 1 at 37 °C for 1 h with shaking at 150 rpm. Untreated APEC cells served as the control. The effect of phage vAPECPW12 on bacterial growth was verified by measuring OD_600_ prior to sample collection. Samples were collected by centrifugation at 6000× *g* for 10 min at 4 °C. After removing the supernatant, the bacterial pellets were washed twice with phosphate-buffered saline (PBS) and resuspended in PBS. The bacterial suspension was placed onto coverslips, and bacterial cells were fixed with 2% (*v*/*v*) glutaraldehyde in 0.1 M PBS at 4 °C for 1 h. After washing twice with PBS, samples were fixed with 1% (*v*/*v*) osmium tetroxide (OsO_4_) in deionized (DI) water for 1 h, followed by three additional PBS washes. The bacterial cells were then dehydrated in a graded series of ice-cold ethanol (20%, 40%, 60%, 80%, and 100%). The coverslips underwent critical point drying with 100% ethanol replaced by liquid CO_2_. The coverslips were coated with gold, and bacterial morphology was visualized using a field-emission SEM (Merlin compact, Zeiss, Jena, Germany) equipped with EDX (Aztec, Oxford, UK) and EBSD (Nordlys Max, Oxford, UK) capabilities.

### 4.13. Phage Stability Under Various Temperatures, pH Values, and UV Light

The stability of *Escherichia* phage vAPECPW12 was assessed under various conditions based on established methods [12,61]. After each condition, samples were serially diluted, and phage titers were measured using the double-layer agar method. For thermal stability, phage suspensions were incubated at −20 °C, 4 °C, 25 °C, 37 °C, 50 °C, 60 °C, 70 °C, and 80 °C for 2 h. To assess pH stability, phage suspensions were prepared in SM buffer adjusted to pH values ranging from 1 to 14 and incubated at 37 °C for 2 h. For UV stability, phage suspensions were placed in open Petri dishes on ice and exposed to UVC light (254 nm) at a distance of 30 cm, with an approximately light intensity of 1390 µW/cm^2^. Samples were collected at 10 min intervals up to 60 min. All experiments were conducted independently in triplicate.

### 4.14. Efficacy of Phage to Remove Preformed Biofilm

The ability of *Escherichia* phage vAPECPW12 to eradicate preformed biofilms was assessed by measuring biofilm biomass and bacterial cell viability in parallel, as described previously [11,12,61,62,63]. Briefly, 100 μL of exponential-phase APEC (OD600 = 0.08) was mixed with 100 μL of TSB and added to flat-bottomed 96-well microtiter plates. The plates were incubated under static conditions at 37 °C for either 24 or 48 h to allow biofilm formation. At the designated time points, the medium was carefully removed, and the wells were gently washed twice with PBS to remove non-adherent cells. Two hundred microliters of phage vAPECPW12, diluted in TSB to various final concentrations (10^5^ to 10^8^ PFU/well), were added to each well and incubated under the same static conditions for 24 h. Biofilms treated with TSB only served as the negative control. Subsequently, the medium in the wells was removed, and the wells were washed twice with PBS. For biofilm biomass quantification, biofilms in the wells were stained with 200 μL of 0.1% crystal violet and incubated in the dark for 30 min. The excess dye was removed, and the wells were washed three times with 300 μL of deionized water and air-dried. The bound dye was solubilized using 200 μL of absolute ethanol, and the absorbance was measured at OD595 using a microplate reader. Each well was filled with 200 μL of TSB and vigorously pipetted to assess bacterial viability within the biofilms. The resulting suspension was serially diluted in TSB and plated on TSA plates. After overnight incubation at 37 °C, bacterial colonies were counted to determine the number of viable cells. For biofilms incubated for 48 h, the medium was replaced with fresh TSB at 24 h post-incubation. All experiments were independently conducted in triplicate.

### 4.15. Ability of Phage to Prevent Biofilm Formation

The efficacy of *Escherichia* phage vAPECPW12 in preventing biofilm formation was evaluated at 24 and 48 h, as described previously [12,61,62,63]. Briefly, 100 μL of phage vAPECPW12 at final concentrations ranging from 10^5^ to 10^8^ PFU/well was added to wells containing 100 μL of APEC (OD600 = 0.08). The mixtures were gently mixed and incubated under standard conditions without shaking at 37 °C for 24 or 48 h. At each time point, the supernatant was removed. The wells were washed twice with 200 μL of PBS, followed by measurement of biofilm biomass and viable cell counts as described above. All experiments were independently conducted in triplicate.

### 4.16. Accession Numbers of the Genome of the Phage

The whole-genome sequence of *Escherichia* phage vAPECPW12 was submitted to the GenBank database under accession number PV874203.

### 4.17. Statistical Analysis

Data analysis was conducted using the GraphPad Prism program, version 10 (GraphPad Software, San Diego, CA, USA, https://www.graphpad.com/scientific-software/prism/, accessed on 4 October 2025). One-way ANOVA with Dunnett’s post hoc test was used to determine statistical significance, with a *p*-value of less than 0.05 considered statistically significant.

## 5. Conclusions

We successfully isolated a novel lytic phage, vAPECPW12, from the genus *Kuravirus*. This phage exhibits lytic activity against multiple APEC isolates and demonstrates stability across a range of temperatures and pH values. It exhibits a high adsorption rate, short latent period, large burst size, potent antibacterial and antibiofilm activity, and lacks undesirable genes, indicating safety for use. These characteristics suggest that phage vAPECPW12 is a promising candidate for phage therapy, biocontrol, or food safety applications. Further research should focus on developing phage cocktails to overcome bacterial resistance to phage and enhance therapeutic efficacy.

## Figures and Tables

**Table 1:** Bacterial lysis efficacy and efficiency of plating (EOP) of *Escherichia* phage vAPECPW12.

Strain	Phage vAPECPW12	
	Lytic Activity	EOP
MDR APEC PW001	+	+
MDR APEC PW002	−	−
MDR APEC PW003	−	−
MDR APEC PW004	−	−
MDR APEC PW005	−	−
MDR APEC PW006	+	Moderate (0.25)
MDR APEC PW007 *	+	High (Host = 1)
MDR APEC PW008	−	−
MDR APEC PW009	+	High (0.86)
MDR APEC PW010	−	−
MDR APEC PW011	+	Moderate (0.25)
MDR APEC PW012	−	−
MDR APEC PW013	+	Low (0.05)
MDR APEC PW014	−	−
MDR APEC PW015	−	−
MDR APEC PW016	−	−
MDR APEC PW017	−	−
MDR APEC PW018	−	−
MDR APEC PW019	+	Moderate (0.46)
MDR APEC PW020	−	−
MDR APEC PW021	−	−
MDR APEC PW022	+	High (0.65)
MDR APEC PW023	−	−
MDR APEC PW024	−	−
MDR APEC PW025	+	Moderate (0.42)
*C. jejuni* PW001	−	−
*C. jejuni* PW002	−	−
*C. jejuni* PW003	−	−
*C. jejuni* PW004	−	−
*C. jejuni* PW005	−	−

Infection results were recorded as +, infection; −, no infection. The asterisk (*) denotes the host strain used for the isolation of *Escherichia* phage vAPECPW12. Efficiency of plating (EOP) was categorized into 4 groups: high production (EOP ≥ 0.5), moderate production (0.1 ≤ EOP < 0.5), low production (0.001 < EOP < 0.1), and no production (EOP ≤ 0.001). All assays were performed independently in triplicate.

## Data Availability

The original contributions presented in this study are included in the article/Supplementary Material. Further inquiries can be directed to the corresponding authors.

## Supplementary Materials

The following supporting information can be downloaded at: https://www.mdpi.com/article/10.3390/ijms262411911/s1.
