# Supplementary material for: Whole-Genome Sequencing and Functional Characterization of a Novel Kuravirus Bacteriophage with Antibiofilm Activity Against Multidrug-Resistant Avian Pathogenic Escherichia coli
Source: Int J Mol Sci. 2025 Dec 10;26(24):11911. doi: 10.3390/ijms262411911 (PMC12733307; doi:10.3390/ijms262411911)

Supplementary Figure S1: Base quality of phage vAPECPW12 genome.

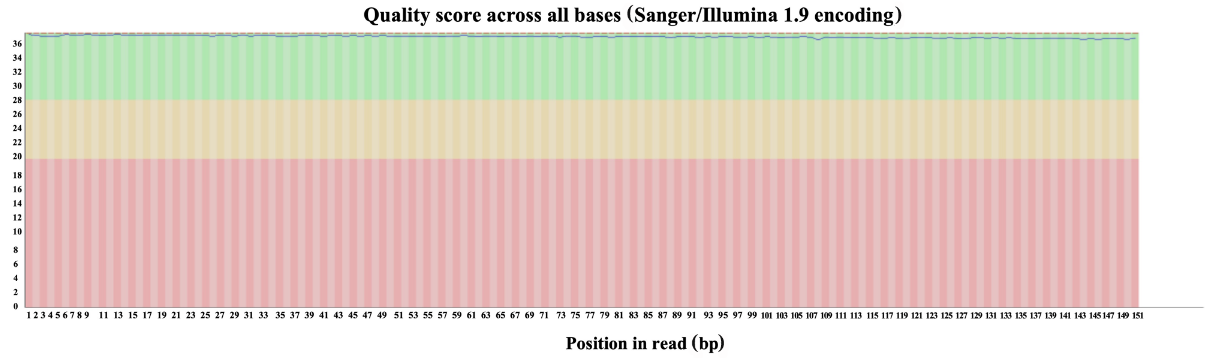

Supplementary Figure S2: Length of the longest contig (a) and cumulative contig plot (b).

a

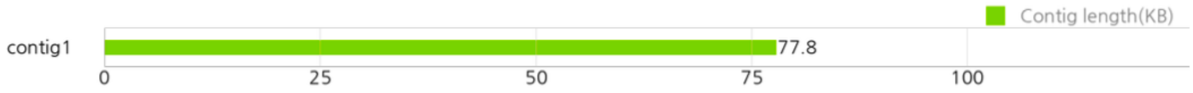

b

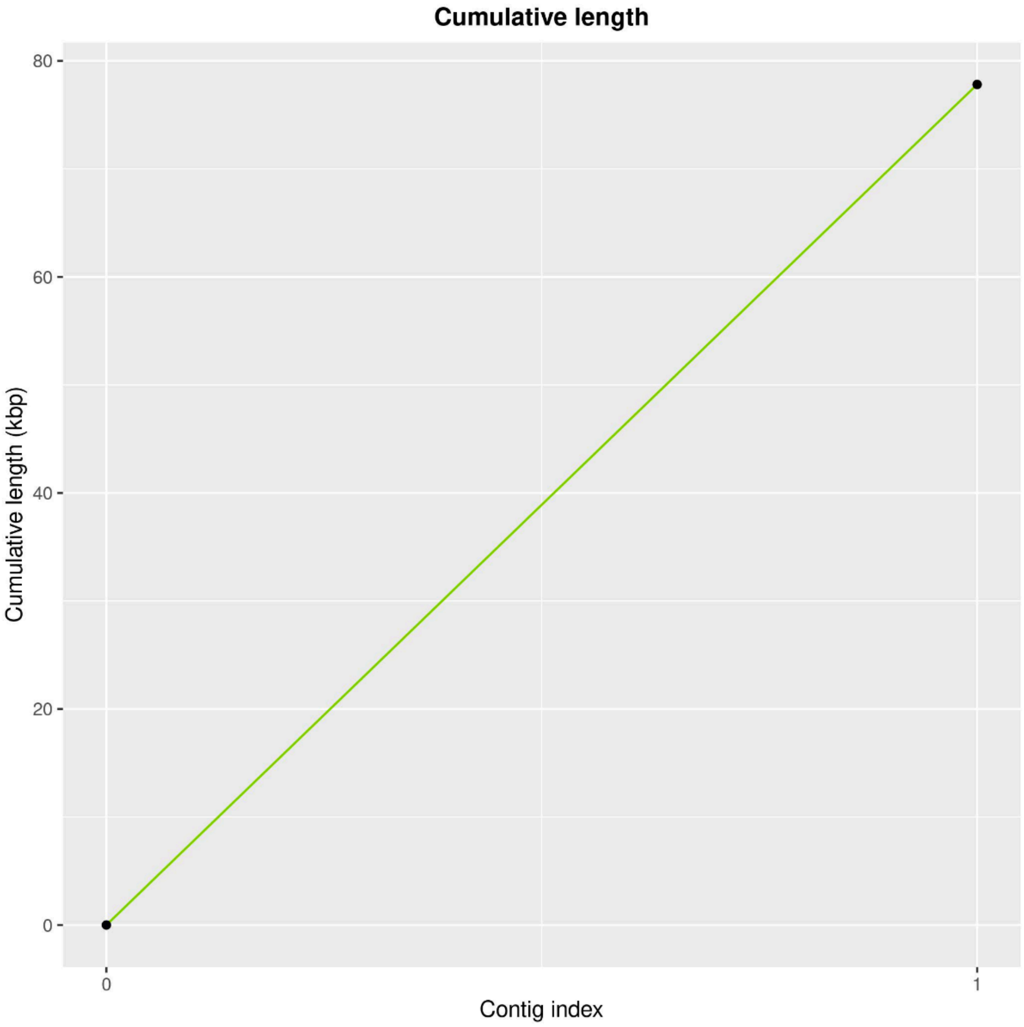

Supplement: Supplementary file 1 [file ijms-26-11911-s001.zip › Supplementary Figures.pdf]
